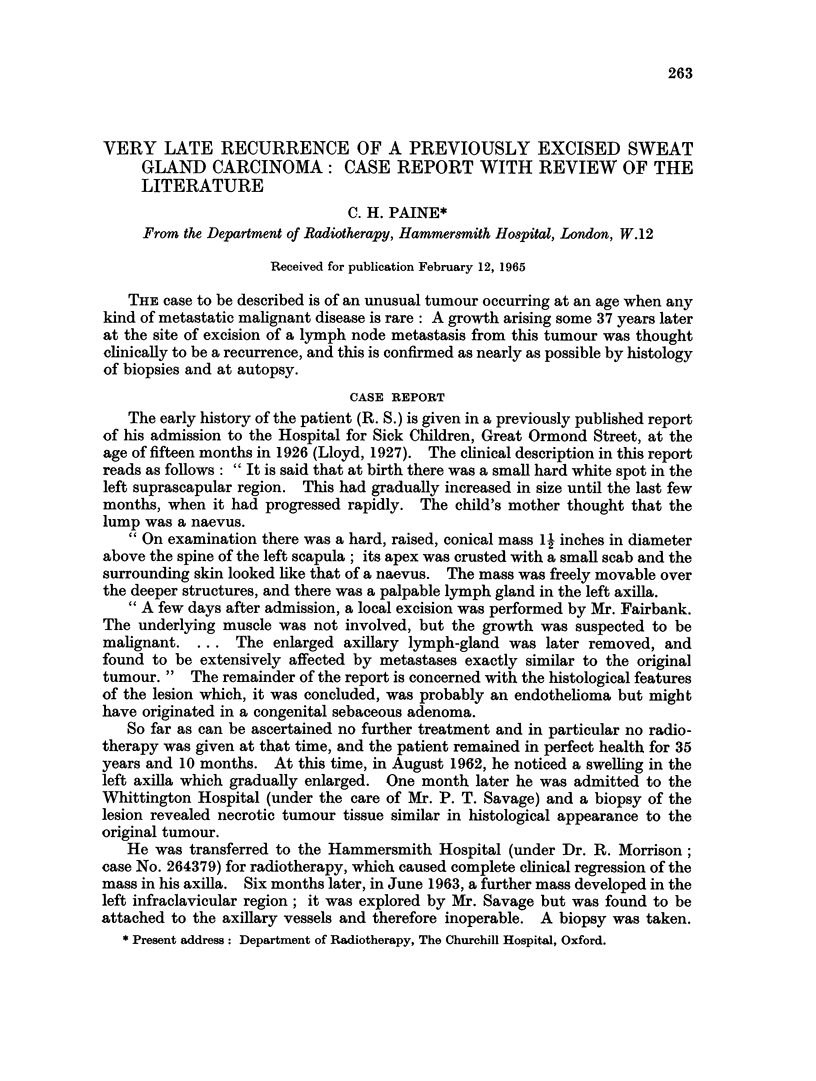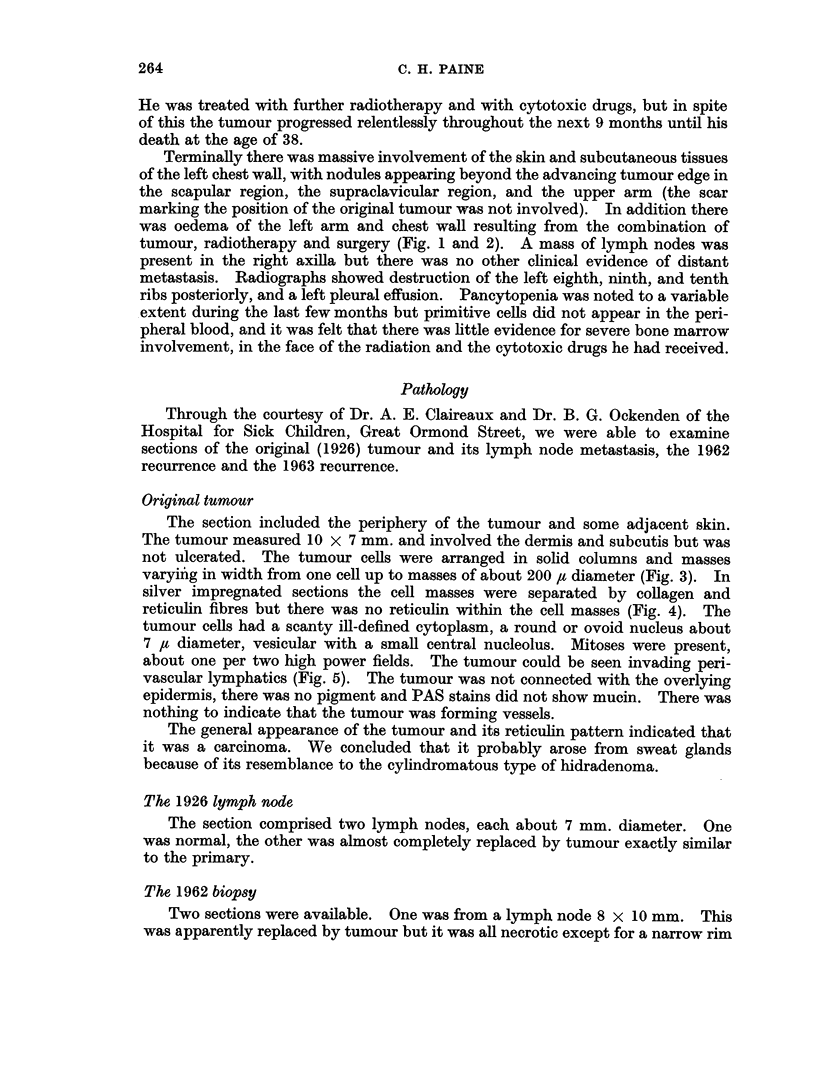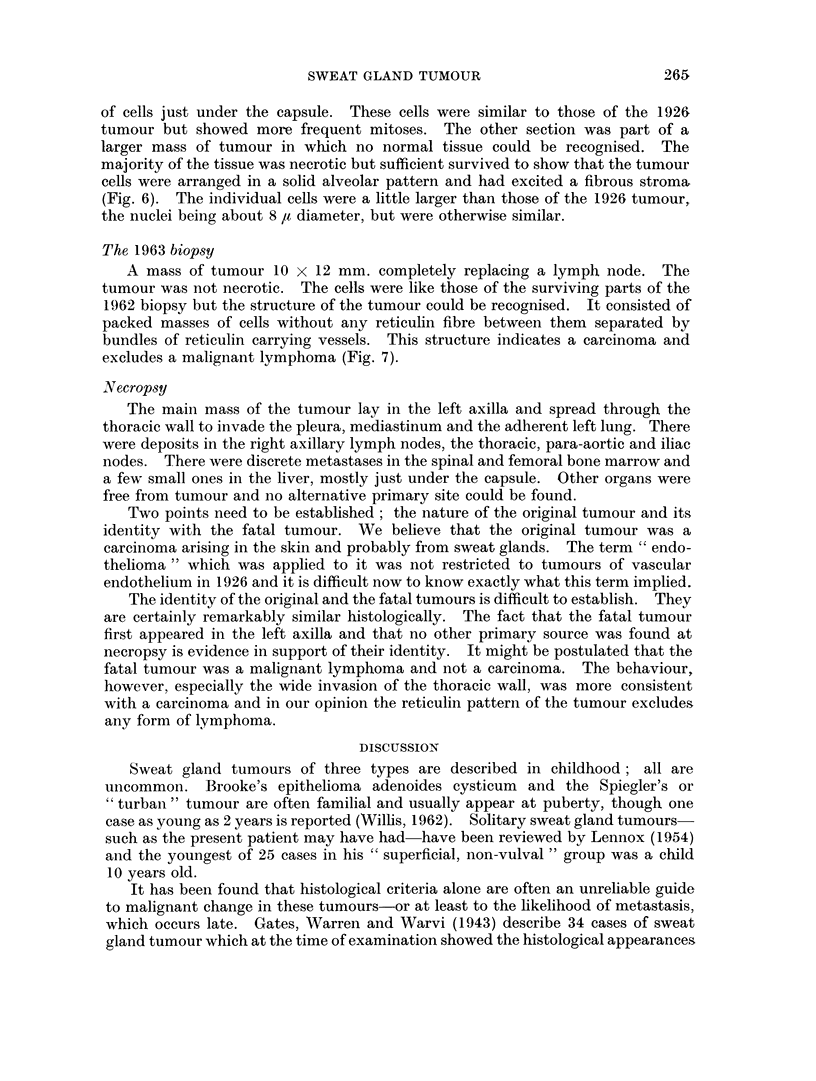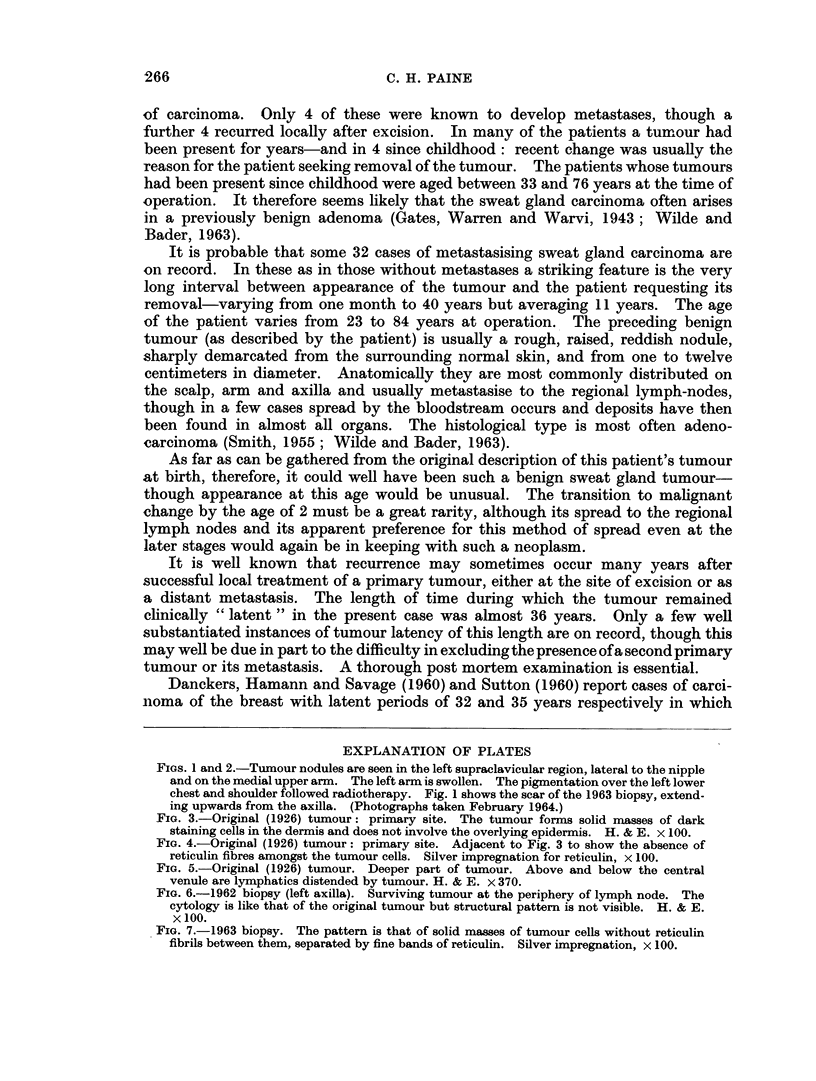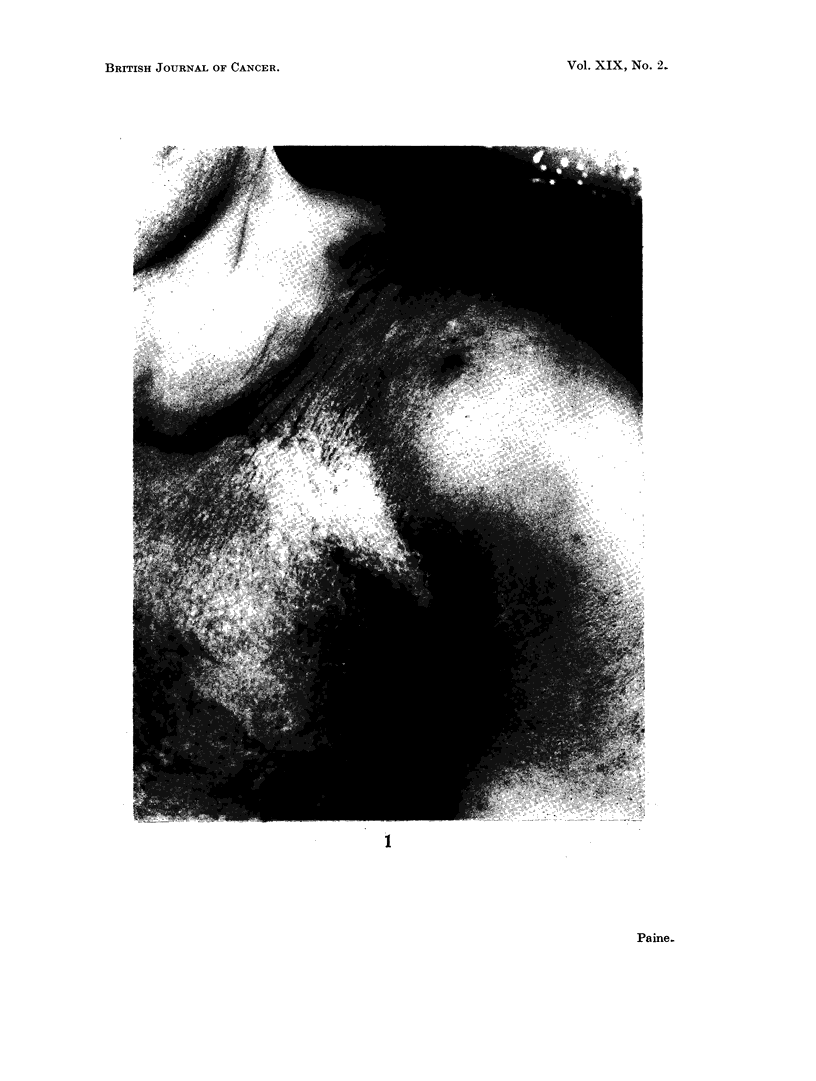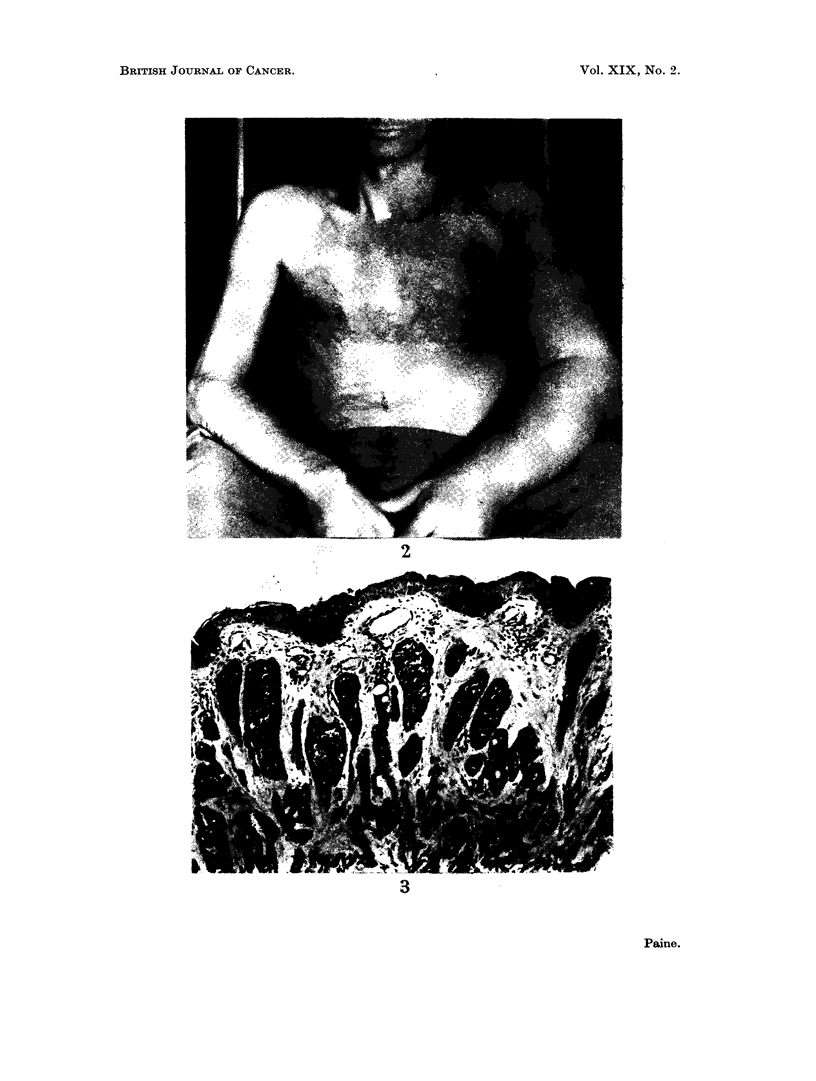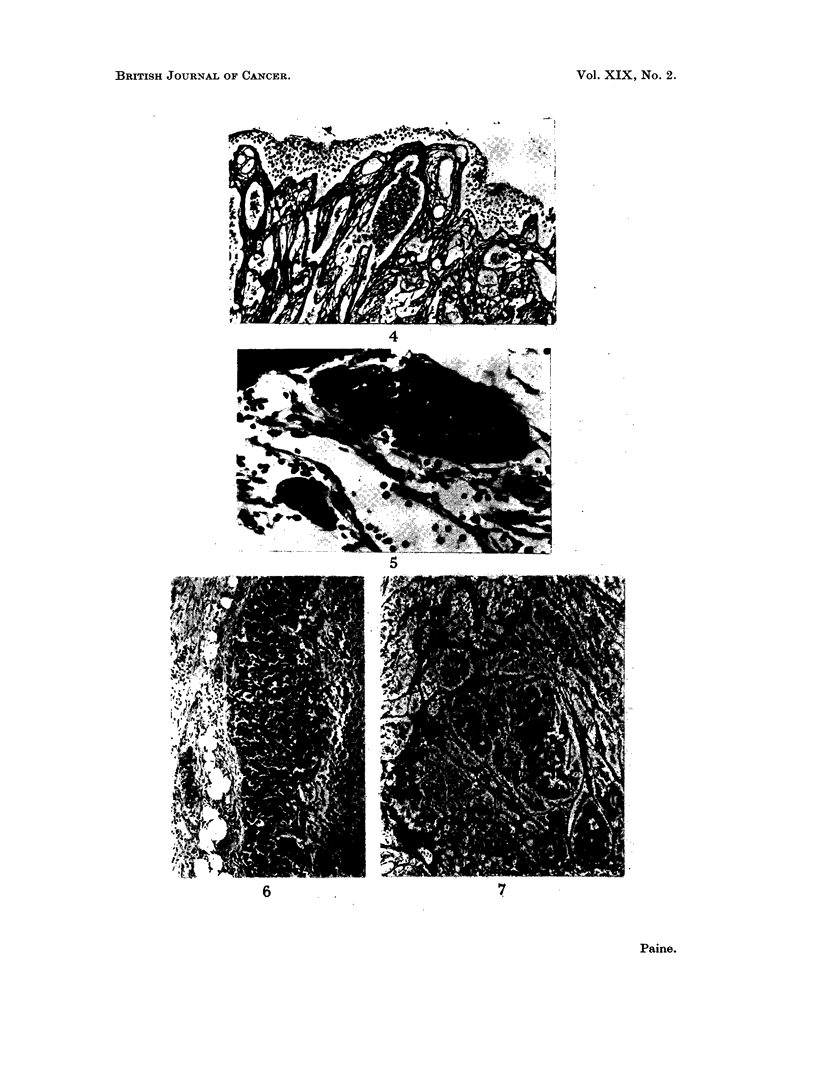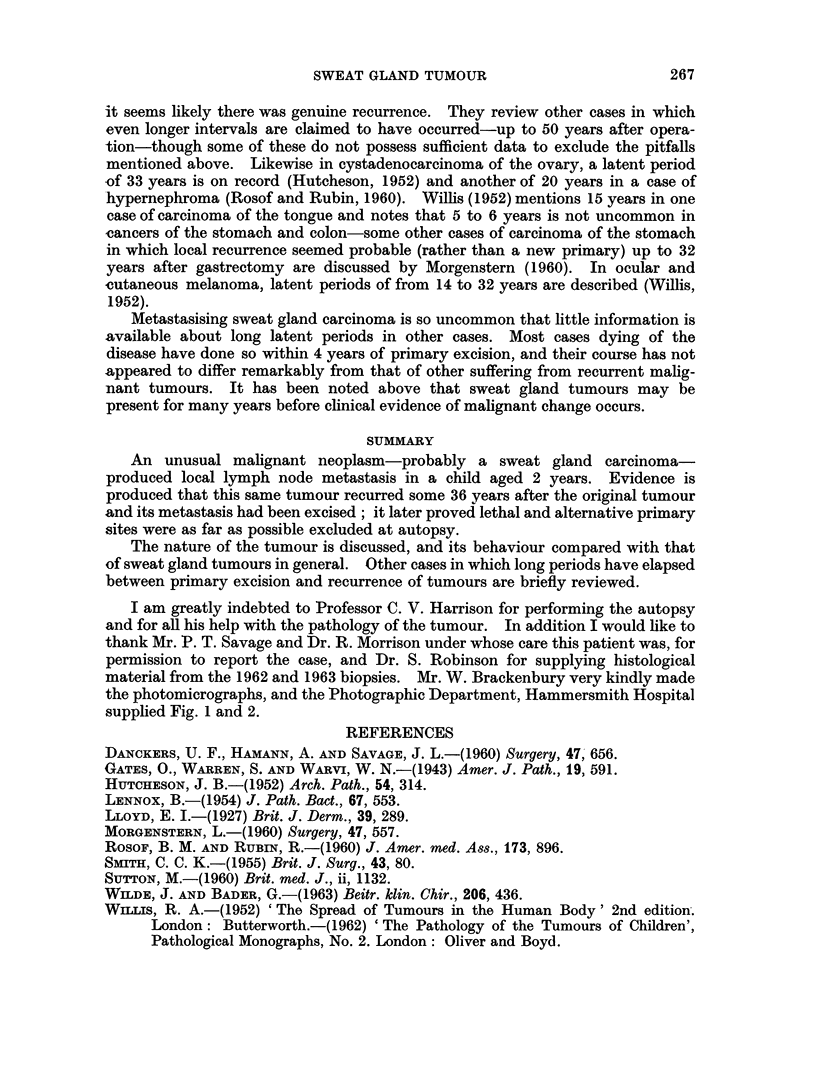# Very Late Recurrence of a Previously Excised Sweat Gland Carcinoma: Case Report with Review of the Literature

**DOI:** 10.1038/bjc.1965.29

**Published:** 1965-06

**Authors:** C. H. Paine

## Abstract

**Images:**


### VERY LATE RECURRENCE OF A PREVIOUSLY EXCISED SWEAT GLAND CARCINOMA: CASE REPORT WITH REVIEW OF THE LITERATURE

C. H. PAINE*

*From the Department of Radiotherapy, Hammersmith Hospital, London, W.12*

Received for publication February 12, 1965

THE case to be described is of an unusual tumour occurring at an age when any kind of metastatic malignant disease is rare : A growth arising some 37 years later at the site of excision of a lymph node metastasis from this tumour was thought clinically to be a recurrence, and this is confirmed as nearly as possible by histology of biopsies and at autopsy.

### CASE REPORT

The early history of the patient (R. S.) is given in a previously published report of his admission to the Hospital for Sick Children, Great Ormond Street, at the age of fifteen months in 1926 (Lloyd, 1927). The clinical description in this report reads as follows : " It is said that at birth there was a small hard white spot in the left suprascapular region. This had gradually increased in size until the last few months, when it had progressed rapidly. The child's mother thought that the lump was a naevus.

" On examination there was a hard, raised, conical mass 1½ inches in diameter above the spine of the left scapula ; its apex was crusted with a small scab and the surrounding skin looked like that of a naevus. The mass was freely movable over the deeper structures, and there was a palpable lymph gland in the left axilla.

" A few days after admission, a local excision was performed by Mr. Fairbank. The underlying muscle was not involved, but the growth was suspected to be malignant. . . . The enlarged axillary lymph-gland was later removed, and found to be extensively affected by metastases exactly similar to the original tumour. " The remainder of the report is concerned with the histological features of the lesion which, it was concluded, was probably an endothelioma but might have originated in a congenital sebaceous adenoma.

So far as can be ascertained no further treatment and in particular no radiotherapy was given at that time, and the patient remained in perfect health for 35 years and 10 months. At this time, in August 1962, he noticed a swelling in the left axilla which gradually enlarged. One month later he was admitted to the Whittington Hospital (under the care of Mr. P. T. Savage) and a biopsy of the lesion revealed necrotic tumour tissue similar in histological appearance to the original tumour.

He was transferred to the Hammersmith Hospital (under Dr. R. Morrison ; case No. 264379) for radiotherapy, which caused complete clinical regression of the mass in his axilla. Six months later, in June 1963, a further mass developed in the left infraclavicular region ; it was explored by Mr. Savage but was found to be attached to the axillary vessels and therefore inoperable. A biopsy was taken.

* Present address : Department of Radiotherapy, The Churchill Hospital, Oxford.

He was treated with further radiotherapy and with cytotoxic drugs, but in spite of this the tumour progressed relentlessly throughout the next 9 months until his death at the age of 38.

Terminally there was massive involvement of the skin and subcutaneous tissues of the left chest wall, with nodules appearing beyond the advancing tumour edge in the scapular region, the supraclavicular region, and the upper arm (the scar marking the position of the original tumour was not involved). In addition there was oedema of the left arm and chest wall resulting from the combination of tumour, radiotherapy and surgery (Fig. 1 and 2). A mass of lymph nodes was present in the right axilla but there was no other clinical evidence of distant metastasis. Radiographs showed destruction of the left eighth, ninth, and tenth ribs posteriorly, and a left pleural effusion. Pancytopenia was noted to a variable extent during the last few months but primitive cells did not appear in the peripheral blood, and it was felt that there was little evidence for severe bone marrow involvement, in the face of the radiation and the cytotoxic drugs he had received.

### *Pathology*

Through the courtesy of Dr. A. E. Claireaux and Dr. B. G. Ockenden of the Hospital for Sick Children, Great Ormond Street, we were able to examine sections of the original (1926) tumour and its lymph node metastasis, the 1962 recurrence and the 1963 recurrence.

### *Original tumour*

The section included the periphery of the tumour and some adjacent skin. The tumour measured 10 × 7 mm. and involved the dermis and subcutis but was not ulcerated. The tumour cells were arranged in solid columns and masses varying in width from one cell up to masses of about 200 $\mu$ diameter (Fig. 3). In silver impregnated sections the cell masses were separated by collagen and reticulin fibres but there was no reticulin within the cell masses (Fig. 4). The tumour cells had a scanty ill-defined cytoplasm, a round or ovoid nucleus about 7 $\mu$ diameter, vesicular with a small central nucleolus. Mitoses were present, about one per two high power fields. The tumour could be seen invading perivascular lymphatics (Fig. 5). The tumour was not connected with the overlying epidermis, there was no pigment and PAS stains did not show mucin. There was nothing to indicate that the tumour was forming vessels.

The general appearance of the tumour and its reticulin pattern indicated that it was a carcinoma. We concluded that it probably arose from sweat glands because of its resemblance to the cylindromatous type of hidradenoma.

### *The* 1926 *lymph node*

The section comprised two lymph nodes, each about 7 mm. diameter. One was normal, the other was almost completely replaced by tumour exactly similar to the primary.

### *The* 1962 *biopsy*

Two sections were available. One was from a lymph node 8 × 10 mm. This was apparently replaced by tumour but it was all necrotic except for a narrow rim of cells just under the capsule. These cells were similar to those of the 1926 tumour but showed more frequent mitoses. The other section was part of a larger mass of tumour in which no normal tissue could be recognised. The majority of the tissue was necrotic but sufficient survived to show that the tumour cells were arranged in a solid alveolar pattern and had excited a fibrous stroma (Fig. 6). The individual cells were a little larger than those of the 1926 tumour, the nuclei being about 8 $\mu$ diameter, but were otherwise similar.

*The* 1963 *biopsy*

A mass of tumour 10 $\times$ 12 mm. completely replacing a lymph node. The tumour was not necrotic. The cells were like those of the surviving parts of the 1962 biopsy but the structure of the tumour could be recognised. It consisted of packed masses of cells without any reticulin fibre between them separated by bundles of reticulin carrying vessels. This structure indicates a carcinoma and excludes a malignant lymphoma (Fig. 7).

*Necropsy*

The main mass of the tumour lay in the left axilla and spread through the thoracic wall to invade the pleura, mediastinum and the adherent left lung. There were deposits in the right axillary lymph nodes, the thoracic, para-aortic and iliac nodes. There were discrete metastases in the spinal and femoral bone marrow and a few small ones in the liver, mostly just under the capsule. Other organs were free from tumour and no alternative primary site could be found.

Two points need to be established ; the nature of the original tumour and its identity with the fatal tumour. We believe that the original tumour was a carcinoma arising in the skin and probably from sweat glands. The term " endothelioma " which was applied to it was not restricted to tumours of vascular endothelium in 1926 and it is difficult now to know exactly what this term implied.

The identity of the original and the fatal tumours is difficult to establish. They are certainly remarkably similar histologically. The fact that the fatal tumour first appeared in the left axilla and that no other primary source was found at necropsy is evidence in support of their identity. It might be postulated that the fatal tumour was a malignant lymphoma and not a carcinoma. The behaviour, however, especially the wide invasion of the thoracic wall, was more consistent with a carcinoma and in our opinion the reticulin pattern of the tumour excludes any form of lymphoma.

### DISCUSSION

Sweat gland tumours of three types are described in childhood ; all are uncommon. Brooke's epithelioma adenoides cysticum and the Spiegler's or " turban " tumour are often familial and usually appear at puberty, though one case as young as 2 years is reported (Willis, 1962). Solitary sweat gland tumours—such as the present patient may have had—have been reviewed by Lennox (1954) and the youngest of 25 cases in his " superficial, non-vulval " group was a child 10 years old.

It has been found that histological criteria alone are often an unreliable guide to malignant change in these tumours—or at least to the likelihood of metastasis, which occurs late. Gates, Warren and Warvi (1943) describe 34 cases of sweat gland tumour which at the time of examination showed the histological appearances of carcinoma. Only 4 of these were known to develop metastases, though a further 4 recurred locally after excision. In many of the patients a tumour had been present for years—and in 4 since childhood : recent change was usually the reason for the patient seeking removal of the tumour. The patients whose tumours had been present since childhood were aged between 33 and 76 years at the time of operation. It therefore seems likely that the sweat gland carcinoma often arises in a previously benign adenoma (Gates, Warren and Warvi, 1943 ; Wilde and Bader, 1963).

It is probable that some 32 cases of metastasising sweat gland carcinoma are on record. In these as in those without metastases a striking feature is the very long interval between appearance of the tumour and the patient requesting its removal—varying from one month to 40 years but averaging 11 years. The age of the patient varies from 23 to 84 years at operation. The preceding benign tumour (as described by the patient) is usually a rough, raised, reddish nodule, sharply demarcated from the surrounding normal skin, and from one to twelve centimeters in diameter. Anatomically they are most commonly distributed on the scalp, arm and axilla and usually metastasise to the regional lymph-nodes, though in a few cases spread by the bloodstream occurs and deposits have then been found in almost all organs. The histological type is most often adenocarcinoma (Smith, 1955 ; Wilde and Bader, 1963).

As far as can be gathered from the original description of this patient's tumour at birth, therefore, it could well have been such a benign sweat gland tumour—though appearance at this age would be unusual. The transition to malignant change by the age of 2 must be a great rarity, although its spread to the regional lymph nodes and its apparent preference for this method of spread even at the later stages would again be in keeping with such a neoplasm.

It is well known that recurrence may sometimes occur many years after successful local treatment of a primary tumour, either at the site of excision or as a distant metastasis. The length of time during which the tumour remained clinically " latent " in the present case was almost 36 years. Only a few well substantiated instances of tumour latency of this length are on record, though this may well be due in part to the difficulty in excluding the presence of a second primary tumour or its metastasis. A thorough post mortem examination is essential.

Danckers, Hamann and Savage (1960) and Sutton (1960) report cases of carcinoma of the breast with latent periods of 32 and 35 years respectively in which

---

### EXPLANATION OF PLATES

FIGS. 1 and 2.—Tumour nodules are seen in the left supraclavicular region, lateral to the nipple and on the medial upper arm. The left arm is swollen. The pigmentation over the left lower chest and shoulder followed radiotherapy. Fig. 1 shows the scar of the 1963 biopsy, extending upwards from the axilla. (Photographs taken February 1964.)

FIG. 3.—Original (1926) tumour : primary site. The tumour forms solid masses of dark staining cells in the dermis and does not involve the overlying epidermis. H. & E. ×100.

FIG. 4.—Original (1926) tumour : primary site. Adjacent to Fig. 3 to show the absence of reticulin fibres amongst the tumour cells. Silver impregnation for reticulin, ×100.

FIG. 5.—Original (1926) tumour. Deeper part of tumour. Above and below the central venule are lymphatics distended by tumour. H. & E. ×370.

FIG. 6.—1962 biopsy (left axilla). Surviving tumour at the periphery of lymph node. The cytology is like that of the original tumour but structural pattern is not visible. H. & E. ×100.

FIG. 7.—1963 biopsy. The pattern is that of solid masses of tumour cells without reticulin fibrils between them, separated by fine bands of reticulin. Silver impregnation, ×100.

1

Paine.

2

3

Paine.

4

5

6

7

it seems likely there was genuine recurrence. They review other cases in which even longer intervals are claimed to have occurred—up to 50 years after operation—though some of these do not possess sufficient data to exclude the pitfalls mentioned above. Likewise in cystadenocarcinoma of the ovary, a latent period of 33 years is on record (Hutcheson, 1952) and another of 20 years in a case of hypernephroma (Rosof and Rubin, 1960). Willis (1952) mentions 15 years in one case of carcinoma of the tongue and notes that 5 to 6 years is not uncommon in cancers of the stomach and colon—some other cases of carcinoma of the stomach in which local recurrence seemed probable (rather than a new primary) up to 32 years after gastrectomy are discussed by Morgenstern (1960). In ocular and cutaneous melanoma, latent periods of from 14 to 32 years are described (Willis, 1952).

Metastasising sweat gland carcinoma is so uncommon that little information is available about long latent periods in other cases. Most cases dying of the disease have done so within 4 years of primary excision, and their course has not appeared to differ remarkably from that of other suffering from recurrent malignant tumours. It has been noted above that sweat gland tumours may be present for many years before clinical evidence of malignant change occurs.

SUMMARY

An unusual malignant neoplasm—probably a sweat gland carcinoma—produced local lymph node metastasis in a child aged 2 years. Evidence is produced that this same tumour recurred some 36 years after the original tumour and its metastasis had been excised ; it later proved lethal and alternative primary sites were as far as possible excluded at autopsy.

The nature of the tumour is discussed, and its behaviour compared with that of sweat gland tumours in general. Other cases in which long periods have elapsed between primary excision and recurrence of tumours are briefly reviewed.

I am greatly indebted to Professor C. V. Harrison for performing the autopsy and for all his help with the pathology of the tumour. In addition I would like to thank Mr. P. T. Savage and Dr. R. Morrison under whose care this patient was, for permission to report the case, and Dr. S. Robinson for supplying histological material from the 1962 and 1963 biopsies. Mr. W. Brackenbury very kindly made the photomicrographs, and the Photographic Department, Hammersmith Hospital supplied Fig. 1 and 2.

### REFERENCES

DANCKERS, U. F., HAMANN, A. AND SAVAGE, J. L.—(1960) *Surgery*, **47**, 656.
GATES, O., WARREN, S. AND WARVI, W. N.—(1943) *Amer. J. Path.*, **19**, 591.
HUTCHESON, J. B.—(1952) *Arch. Path.*, **54**, 314.
LENNOX, B.—(1954) *J. Path. Bact.*, **67**, 553.
LLOYD, E. I.—(1927) *Brit. J. Derm.*, **39**, 289.
MORGENSTERN, L.—(1960) *Surgery*, **47**, 557.
ROSOF, B. M. AND RUBIN, R.—(1960) *J. Amer. med. Ass.*, **173**, 896.
SMITH, C. C. K.—(1955) *Brit. J. Surg.*, **43**, 80.
SUTTON, M.—(1960) *Brit. med. J.*, ii, 1132.
WILDE, J. AND BADER, G.—(1963) *Beitr. klin. Chir.*, **206**, 436.
WILLIS, R. A.—(1952) ‘The Spread of Tumours in the Human Body’ 2nd edition. London : Butterworth.—(1962) ‘The Pathology of the Tumours of Children’, Pathological Monographs, No. 2. London : Oliver and Boyd.